# Shooting Your Accuracy in the Foot? Examining the Short-Term Effect of Playing an Action or Strategy Video Game on Cognitive Control

**DOI:** 10.3390/ijerph18158001

**Published:** 2021-07-28

**Authors:** Michaela Rice, Alexis Lease, MaLia Walker, Kira Bailey

**Affiliations:** David O. Robbins Neuroscience Program, Psychology Department, Ohio Wesleyan University, Delaware, OH 43015, USA; mickeyarroz@gmail.com (M.R.); alexisnlease@gmail.com (A.L.); maliawalker97@gmail.com (M.W.)

**Keywords:** video games, cognitive control, ERPs

## Abstract

The current study examined the effects of brief video game exposure on cognitive control using event-related potentials (ERPs). Cognitive control was assessed by ERP components associated with the ability to detect (N2) and resolve (SP) conflict when the conflict was either expected or unexpected. After playing either an action or strategy video game, participants completed a counting Stroop task while ERPs were recorded. The proportion of congruent to incongruent trials was manipulated across blocks to create conditions where conflict was expected or unexpected. While visual inspection of the behavioral and neural data revealed interesting patterns by video game, none of those effects were statistically significant. This is consistent with some previous work and inconsistent with other published data, suggesting that there is still much to learn about the relationship between cognitive control and video game experience.

## 1. Introduction

Video gaming has become a prevalent aspect of the United States’ culture [1]. According to a report by the Entertainment Software Association [1], during the year of 2018, 65% of American adults played video games, 70% of American families had at least one child who played video games, and 21% of gamers were under the age of 18. A study in 2018 showed that “gamers” in the US play an average of six hours of video games per week [2]. Furthermore, approximately 48% of video game sales are of action/shooter games [1]. For these reasons, it is critical to understand the potential effects that video gaming may have on our cognitive capabilities, as these media are being rapidly consumed.

The purpose of the current study was to investigate how short-term exposure of a video game, either first-person shooter (FPS) or strategy, could influence the neural correlates of cognitive control. Cognitive control is the ability to manage, adjust, and order thoughts and planned actions in congruence with internal goals [3]. Two important aspects of cognitive control are monitoring and resolving conflict. Monitoring conflict is the process of detecting errors; it is associated with transient activity in the prefrontal cortex and anterior cingulate cortex [3]. Resolving conflict is the process of finding a solution or correcting an error; it is associated with sustained activity in the parietal cortex and lateral prefrontal cortex [3]. These aspects of cognitive control were elicited and measured in this study via the counting-Stroop (CStroop) and event-related potentials (ERPs) after participants had played an FPS or strategy video game for 20 min.

### 1.1. Video Game Effects

Researchers have investigated the effects of video games for over twenty years now, but questions remain regarding which games elicit which effects. Comparisons across studies can be hindered by the fact that there are various different approaches to studying video game effects on cognitive control, including genre (e.g., action, strategy), length of exposure (e.g., short or long-term), and type of assessment (e.g., behavioral, neural). For the most part, previous studies have largely investigated the effects of long-term video game exposure on cognitive control via video game training methods or in individuals who have high levels of video game experience. One of the biggest gaps in the literature is the short-term effects of video game exposure on cognitive control in the brain. To help fill this gap and expand the field’s knowledge of two of the most-played video game genres [1], the current study examines the short-term effects of FPS and action games on the neural correlates of cognitive control. Cognitive control encompasses various different processes, any of which could be impacted by video game experience, as recently reviewed by Bavelier and Green [4]. In the previous literature, at least one study found no differences in cognitive control, as measured by memory, attention, and executive function cognitive tasks, between the two genres after 20 h of video game play [5]. In contrast, a number of studies have found positive and negative effects on cognitive control from both game genres [6,7,8,9], some of which will be highlighted in the following paragraphs.

Training on a strategy game for 20 h has been shown to significantly improve performance on cognitive tasks related to attention, as indexed by the P3 ERP component and delta EEG waves [6]. Another study demonstrated that during 50 min of strategy game play, the binding potential of [^11^C]RAC, a radioligand that binds to dopamine receptors, was reduced, especially in the ventral striatum [10]. This indicates an increase in dopamine concentration during video game play. As there was an increase in the reduction in the binding of [^11^C]RAC, the participants performed better and advanced to more levels. Any changes observed in the dorsal striatum could be associated with sensorimotor coordination and response selection, since it receives input from motor, sensory, premotor, and dorsal prefrontal cortices. Researchers have theorized that dopaminergic release may be related to learning, reinforcement, attention, and sensorimotor integration, so the increase in dopamine concentration in the ventral striatum during video gaming and the increased success in performance could support the idea that all of these processes are supported and utilized during gaming [10]. A meta-analysis of video gaming and cognitive functions indicated that high levels of experience in strategy games, defined as anywhere from 8 to 21 h of video game training, is significantly associated with the most positive effects on cognitive control in comparison to other games [11].

In contrast, only one study has demonstrated negative effects of strategy game exposure [7]. In this study, the effects of exergaming, traditional video gaming, and aerobic exercise on cognitive control were compared. A strategy video game was used in the traditional video gaming condition, with exposure lasting 20 min. Neither exergaming nor video gaming served to improve cognitive control, especially in comparison to aerobic exercise. These results suggest that strategy video gaming does not elicit any changes in the allocation of attention, inhibition, or other mechanisms of cognitive control [7]. This study did not compare the effects of a strategy game to an FPS game, so it is unclear how these results would compare to other game genres. Overall, the greater number of studies demonstrating positive effects of strategy video games, as opposed to negative effects, indicates that exposure to a strategy video game is more likely to be associated with improved cognitive control.

There are also a number of studies demonstrating the positive effects of FPS/action games. A study by Salminen and Ravaja [8] demonstrated an increase in occipital theta activation during one hour of gameplay of an action game. Increased theta activity has been associated with increased concentration and the processing of emotional information, so these results could indicate an increase in cognitive functioning during game play for this duration [8]. Another study by Chandra, Sharma, Salam, Jha, and Mittal [12] has demonstrated improved reaction times, processing speeds, and reduced stress levels from training for 50 h in an action video game. Finally, another study has shown that training in an FPS game for ten hours can lead to the enhancement of top-down spatial selective attention, as demonstrated by improved performance on an attentional visual field task [13].

A greater number of studies demonstrate negative effects of playing FPS/action video games. A study by Wang et al. [14] has shown that an hour of playing an action video game can lead to lower accuracy in a counting-Stroop task in comparison to a non-action video game. Additionally, exposure to the action video game for an hour can lead to reduced activity in the prefrontal cortex, specifically in the left dorsolateral prefrontal cortex and the dorsal anterior cingulate cortex [14]. These regions are significant for executive and cognitive control processing such as inhibition and selective attention, so decreased activity in these areas indicates a decrease in executive and cognitive functioning [14]. Another study found that high levels of action video game experience, with an average of 43 h of gameplay per week, are associated with a decrease in some aspects of cognitive control in the color-word Stroop task, as indicated by the medial frontal negativity and frontal slow wave ERPs [9]. A study by Kowal, Toth, Exton, and Campbell [15] demonstrated that participants who played action video games for anywhere between 1 and 23 h per week performed significantly faster on the Stroop and Trail-Making tests than non-video gamers, but they also made significantly more errors, suggesting that action video gamers prefer speed over accuracy in their cognitive functioning [15]. Finally, Hummer, Kronenburger, Wang, and Mathews [16] studied how up to 14 h of action video game exposure would influence inhibition by having participants perform a go/no-go task before and after a week of action video game exposure. The results indicated that 7 to 14 h of exposure to the action video game led to decreases in inhibition-related activity of the inferior frontal gyrus and cerebellum, as well as an increase in self-reports of issues with executive functioning. This study suggests that high levels of exposure to an action video game can impair inhibition and negatively impact cognitive control [16]. The greater number of negative effects of FPS/action video game exposure, as opposed to positive effects, indicates that exposure to an FPS/action video game is more likely to be associated with worsened cognitive control.

### 1.2. The Current Study

The current study focuses on two features of cognitive control utilized with the CStroop task: conflict detection and conflict resolution. In this task, participants were instructed to respond to the number of digits presented rather than the identity of the digits. Like the traditional color-word Stroop [17], participants encounter congruent (e.g., 1, 22, and 4444) and incongruent trials (e.g., 11, 2, 444). Participants experience greater conflict on the incongruent trials, and an individual’s response to conflict can shift based on current goals or environment, for example, whether the conflict is expected or unexpected. To influence participants’ expectation of conflict, a proportion congruent manipulation was utilized, in which one block of trials was 75% congruent (i.e., low expectation of conflict) and the other block of trials was 75% incongruent (i.e., high expectation of conflict). Previous work with the proportion-congruent manipulations reveals that participants are better able to detect and resolve conflict when they expect to encounter it more frequently [18].

The ERPs utilized in this study were the N2 and the conflict sustained potential (SP). The N2 is characterized by negative amplitude arising over the mid-frontal region of the scalp from the detection of an aberrant stimulus in a set of standard stimuli. It is typically observed 180 to 325 ms proceeding the presentation of the stimulus [19]. The N2 is believed to be associated with conflict detection and identification [20]. The SP is characterized by sustained positivity over the central-parietal region of the scalp, with a larger amplitude observed in incongruent trials in comparison to congruent trials [18,21]. It is evoked 500–1000 ms after the stimulus has been presented [18]. The SP distinguishes responses to congruent and incongruent trials, indicating its association with conflict resolution.

As mentioned previously, one of the biggest gaps in the literature is the short-term effects (i.e., 10 min to 1 h) of video game exposure on cognitive control in the brain. While many researchers have examined the short-term effects of video games on other characteristics, such as aggression, most of the work on cognition looks at the effects of several hours of play. In order to examine whether there are immediate effects of short-term exposure to a video game on two aspects of cognitive control, conflict detection and conflict resolution, participants were randomly assigned to play either a strategy game, StarCraft (SC), or a first-person shooter game, Unreal Tournament 3 (UT), for 20 min. After video game exposure, participants completed the CStroop while electroencephalography (EEG) was recorded. The N2 and SP ERP components were analyzed offline. Based on the previous research with FPS/action video games, it was hypothesized that exposure to UT would result in lower accuracy, particularly on incongruent trials, in the CStroop. Additionally, it was hypothesized that exposure to UT would increase the amplitude of the N2, indicating greater experience of conflict on incongruent trials, and decrease the amplitude of the SP, indicating worse conflict resolution. If exposure to strategy video games improves cognition as some previous studies suggest, then exposure to SC was expected to improve accuracy, decrease the amplitude of the N2, and increase the amplitude of the SP, demonstrating better resolution of conflict.

## 2. Materials and Methods

### 2.1. Participants

Forty undergraduate students were recruited from a small mid-western university through Introduction to Psychology classes and flyers on campus. Ages of the participants ranged from 17 to 24 (*M =* 19.11, *SD* = 1.39). Five participants were excluded due to an excess of artifacts in their EEG data, making the final sample size 35. Fifty-seven percent of the participants were female; 14.3% of the participants were left-handed. Written informed consent was obtained from each participant. Participants enrolled in Introduction to Psychology received course credit for participation; all other participants were paid USD 15 for their time.

### 2.2. Materials

Internet Gaming Disorder Scale [22]: Participants reported any present symptoms of Internet Gaming Disorder (IGD). Participants responded “yes” or “no” to several statements that tapped into elements of the disorder (e.g., “have there been periods when all you could think of was the moment that you could play a game?”). Answering yes to five statements was indicative of IGD.

ADHD Measure: Participants reported the frequency of potential ADHD symptoms by indicating “never”, “rarely”, “sometimes”, “often”, or “very often” to a series of questions (e.g., “How often do you have trouble wrapping up the fine details of a project, once the challenging parts have been done?”).

Barratt Impulsiveness Scale-11 (BIS-11): Participants reported their impulsive thoughts and behaviors by answering 1, 2, 3, or 4, indicating “Rarely/Never”, “Occasionally”, “Often”, or “Almost Always/Always”, respectively, to a series of statements (e.g., “I plan tasks carefully”).

Video Game Experience: Participants indicated their previous video game experience by reporting the number of hours they spent playing video games during weekdays and weekends. Participants indicated the genre of games most frequently played by answering “1: I never play it”, “2: I rarely play it”, “3: I occasionally play it”, “4: I sometimes play it”, “5: I often play it”, “6: I always play it” to a series of statements (e.g., “Sports”, “Action/Adventure”, “Puzzle Games”).

Video Games: Two commercial video games were utilized in this study, *StarCraft* by Blizzard Entertainment (Irvine, CA, USA) and *Unreal Tournament 3* by Epic Games (Cary, NC, USA). *StarCraft* is a real-time strategy video game set on a futuristic planet where players must compete with other players or non-player character (NPC) forces, making careful decisions about how to best use their resources to create additional buildings and fight enemy combatants. *Unreal Tournament 3* is a first-person shooter video game; the current study utilized the “deathmatch” mode in which the player must kill all other players to be the last one standing in order to win the round. Both games involve some direct combat with other characters. *StarCraft* generally requires more thoughtful planning to be successful, being slower paced with bursts of more intense action. *Unreal Tournament 3* is typically more fast-paced action. In the current study, participants played against NPCs in both games.

Counting-Stroop: The task was constructed using E-Prime. Participants used keys “1”, “2”, “3”, and “4” on the keyboard during the task to indicate the number of digits present. On congruent trials, the number of digits matched the identity of the digits (1, 22, 333, 4444), and on incongruent trials the number of digits did not match the identity of the digits (2, 33, 444, 1111, etc.). The numbers were presented in white, Arial, size 24 font on a black background.

The practice session contained 24 trials, with half of the trials incongruent. Each trial was followed by feedback indicating either “correct” or “incorrect”. The response–stimulus interval (RSI) was 500 ms. The testing session had 4 blocks of trials, with 2 blocks as Mostly congruent (Mc) and 2 blocks as Mostly incongruent (Mi). Each block contained 96 trials. The RSI was 1000 ms. The Mc blocks had 75% congruent (C) trials, 25% incongruent (I) trials, and the Mi blocks had 75% I trials, 25% C trials. There were four conditions that were labeled based on their block/trial configuration (Mc or Mi, C or I). Mc/I was defined as unexpected conflict and Mi/I was defined as expected conflict.

### 2.3. Procedures

Participants gave informed consent and then completed the questionnaires. The EEG cap was then applied. Participants played 20 min of either SC (*n* = 18) or UT (*n* = 17) based on random assignment. Following gameplay, participants completed the cognitive tasks, the CStroop and Flanker. For the scope of this paper, only the CStroop data were analyzed and discussed. The order of the tasks was counterbalanced, as well as the order of Mc and Mi blocks. ERPs were recorded during completion of the cognitive tasks. After completing the cognitive tasks, participants were provided with supplies to wash their face and hair. The researchers debriefed the participants and compensated them for their participation.

### 2.4. EEG Recording and Analysis

The EEG (bandpass 0.02–150 Hz, sampling rate 500 Hz) was recorded from an array of 32 actiCap active electrodes based on an extended 10–20 system using actiCHamp and Pycorder software (Brain Vision, LLC, Morrisville, NC, USA). During recording, all electrodes were referenced to electrode Cz. For analysis, the EEG data were re-referenced to an average reference and a 0.1–20 Hz zero-phase-shift bandpass filter was applied. Data were analyzed in MatLab using EEGLab with the ERPLab plug-in. Ocular artifacts were corrected using the Independent Component Analysis feature in EEGlab. The ERP epoch included −200 to 1000 ms of activity around stimulus onset. ERPs were averaged for trials associated with correct responses where response time was less than 5000 ms.

The amplitude of the N2 was measured as mean voltage between 250 and 350 ms after stimulus onset at electrodes F3, Fz, and F4. The selection of these electrodes was based on visual inspection of the data and on the distribution of the N2 reported in prior research [18,19,21]. The amplitude of the SP was measured as mean voltage between 600 and 800 ms after stimulus onset at electrodes P3, Pz, and P4. These or similar electrodes have been used in previous studies to measure the conflict SP [18,21,23,24]. Mean differences in ERP amplitude were evaluated using analysis of variance (ANOVA) with the Huynh–Feldt [25] corrected degrees of freedom when necessary.

## 3. Results

### 3.1. Questionnaires

Participants who played SC (*M* = 11.7 h per week, *SE* = 2.95) and UT (*M* = 12.2 h per week, *SE* = 2.91) did not significantly differ in video game experience, *t*(33) = 0.13, *p* = 0.90, *d* = 0.04. Likewise, scores on the IGD did not differ between groups (SC: *M* = 1.3, *SE* = 1.46; UT: *M* = 1.41, *SE* = 0.94), *t*(33) = 0.19, *p* = 0.85, *d* = 0.07. Group differences for self-reports on ADHD were marginally significant, with participants who played SC (*M* = 12.33, *SE* = 0.74) reporting higher scores than those who played UT (*M* = 10.24, *SE* = 0.76), *t*(33) = 1.98, *p* = 0.06, *d* = 0.67. A similar pattern was observed for the BIS-11, with SC participants (*M* = 71.83, *SE* = 1.53) scoring marginally higher than UT participants (*M* = 67.24, *SE* = 1.97), *t*(33) = 1.86, *p* = 0.07, *d* = 0.63.

### 3.2. Behavioral

**Figure 1:**
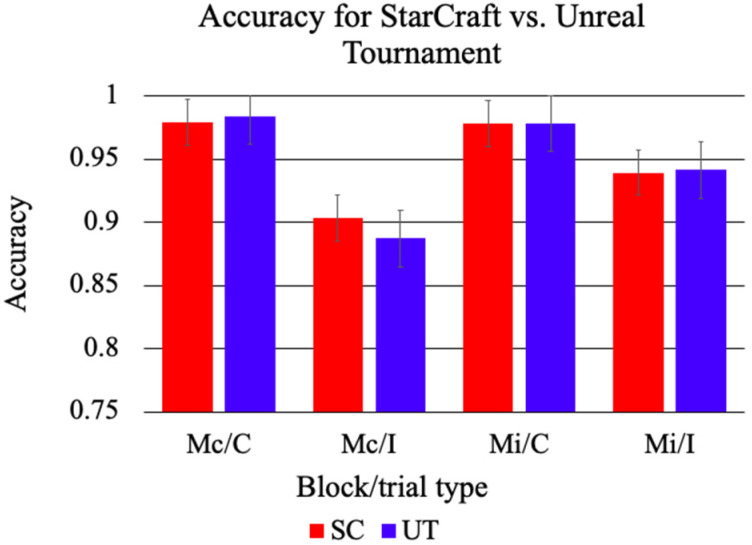
Mean accuracy in the CStroop for SC and UT. The participants in both gaming groups were less accurate for I trials in comparison to C trials. I trials were more accurate in the Mi block than the Mc block. There were no significant differences.

Accuracy and response time (RT) were analyzed using separate 2 Game (UT, SC) × 2 Block (Mc, Mi) × 2 Congruency (C, I) ANOVAs. For accuracy, there were main effects for Block and Congruency. Accuracy was lower in the Mc Block (*M* = 0.94, *SE* = 0.01) compared to the Mi Block (*M* = 0.97, *SE* = 0.00), *F*(1, 33) = 17.55, *p* < 0.001, *η*^2^ = 0.12. C trials had a higher mean accuracy (*M* = 0.99, *SE* = 0.00) than I trials (*M* = 0.92, *SE* = 0.01), *F*(1, 33) = 98.50, *p* < 0.001, *η*^2^ = 0.47. There was a significant two-way interaction effect for Block by Congruency, indicating that participants responded with different levels of accuracy across the two blocks, *F*(1, 33) = 32.64, *p* < 0.001, *η*^2^ = 0.14. Post hoc analyses revealed that C trials did not differ by block, *F*(1, 33) = 0.37, *p* = 0.55. However, I trials were more accurate in the Mi (*M* = 0.95, *SE* = 0.01) block in comparison to the Mc block (*M* = 0.90, *SE* = 0.01), *F*(1, 33) = 29.09, *p* < *0*.001 (see Figure 1). All other main effects and interactions were non-significant, all *F*s < 2.69, all *p*s > 0.11.

**Figure 2:**
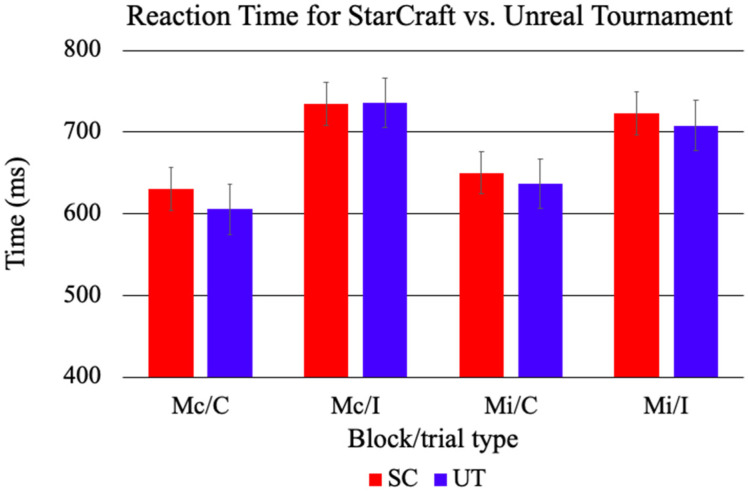
Mean reaction times in the CStroop for SC and UT. The participants for both UT and SC had slower reaction times in I trials in comparison to C trials. I trials were responded to faster in the Mi block and C trials were responded to slower in the Mi block in comparison to the Mc block. There were no significant differences.

For RT, there was a significant main effect of Congruency. C trials had a shorter mean reaction time (*M* = 634 ms, *SE* = 19) than I trials (*M* = 730 ms, *SE* = 22), *F*(1, 33) = 183.19, *p* < 0.001, *η*^2^ = 0.14. There was a significant two-way interaction effect for Block by Congruency, indicating that the participants’ reaction time was influenced by the block type, *F*(1, 33) = 14.72, *p* < 0.001, *η*^2^ = 0.01. Post hoc analyses revealed that participants responded significantly slower on C trials in the Mi block (*M* = 646 ms, *SE* = 22) in comparison to the Mc block (*M* = 622 ms, *SE* = 18), *F*(1, 33) = 5.22, *p* = 0.03. Furthermore, participants responded significantly faster on I trials in the Mi block (*M* = 717 ms, *SE* = 22) in comparison to the Mc block (*M* = 743 ms, *SE* = 23), *F*(1, 33) = 4.76, *p* = 0.04 (see Figure 2). All other main effects and interactions were non-significant, all *F*s < 1.47, all *p*s > 0.23.

### 3.3. ERPs

#### 3.3.1. N2

**Figure 3:**
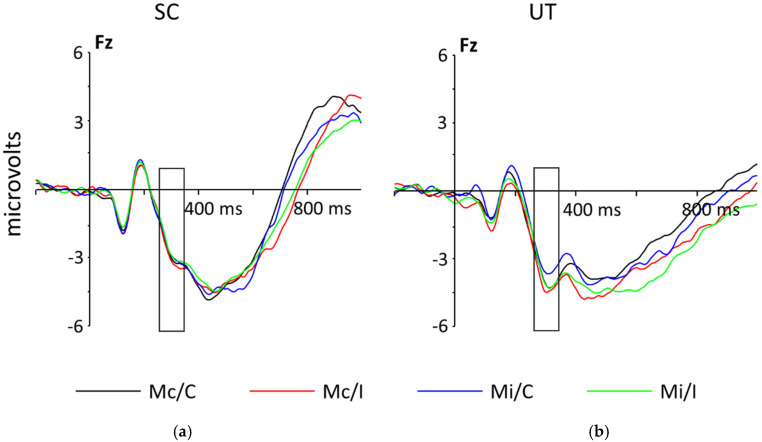
Grand-averaged stimulus-locked ERPs at electrodes Fz demonstrating the time course of the N2 for (**a**) participants who played SC and (**b**) participants who played UT. The UT group shows a slight pattern of I trials being more negative in both blocks, but overall, there were no significant differences between the gaming groups.

The N2 was analyzed in a 2 Game (UT, SC) × 2 Block (Mc, Mi) × 2 Congruency (C, I) × 3 Electrode (F3, Fz, F4) ANOVA. The main effect of Electrode was significant, *F*(1, 33) = 31.29, *p* < *0*.001, *η*^2^ = 0.02. Post hoc analyses demonstrated that F3 (*M =* −4.35 μV, *SE* = 0.43) and Fz (*M =* −4.29 μV, *SE* = 0.53) were not statistically different from each other, *F*(1, 33) = 0.06, *p* = 0.81. However, both F3 and Fz (*M =* −4.32 μV, *SE* = 0.47) were statistically different from F4 (*M* = −3.39 μV, *SE* = 0.52), *F*(1, 33) = 31.29, *p* < *0*.001. This indicates that the N2 was larger in the electrodes over the midline (Fz) and left side of the scalp (F3) than the right side (F4). Visual inspection of the N2 revealed a moderately larger peak for UT (*M =* −4.15 μV, *SE* = 0.69) than for SC (*M =* −3.87 μV, *SE* = 0.67), but this was not significant, *F*(1, 33) = 0.08, *p* = 0.78, *η*^2^ = 0.002 (see Figure 3). All other main effects and interactions were non-significant, all *F*s < 1.77, all *p*s > 0.19.

#### 3.3.2. SP

**Figure 4:**
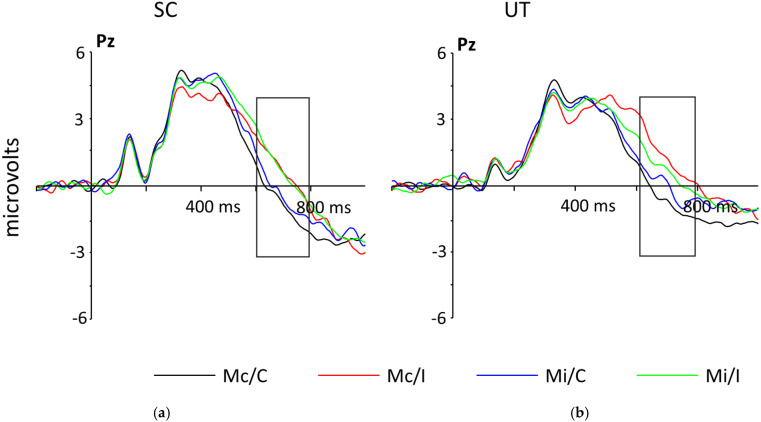
Grand-averaged stimulus-locked ERPs at electrodes Pz demonstrating the time course of the SP for (**a**) participants who played SC and (**b**) participants who played UT. I trials are significantly more positive than C trials, with the greatest amplitude in the Mc block. There were no significant differences between the two gaming groups.

The SP was analyzed in a 2 Game (UT, SC) × 2 Block (Mc, Mi) × 2 Congruency (C, I) × 3 Electrode (P3, Pz, P4) ANOVA. The main effect for Congruency was significant, with mean neural response for the I trials more positive (*M* = 0.91 μV, *SE* = 0.55) than C trials (*M =* −0.32 μV, *SE* = 0.45), *F*(1,33) = 33.03, *p* < 0.001, *η*^2^ = 0.04, indicating a stronger neural response to the conflict in the task. The Block by Congruency interaction was significant, *F*(1,33) = 7.24, *p* = 0.01, *η*^2^ = 0.01, as reflected by the difference between C and I trials being significantly larger in the Mc block (*M =* 1.69 μV, *SE* = 0.31) than the Mi block (*M* = 0.77 μV, *SE* = 0.23), *F*(1, 33) = 7.24, *p* = 0.11 (see Figure 4). Taken together, these results demonstrate that conflict in the task evoked a stronger neural response than non-conflict for both video gaming groups, with the Mc block eliciting the strongest response to conflict, indicating an effect of the task. The SP had a slightly larger peak for UT (*M* = 0.70 μV, *SE* = 0.70) than for SC (*M* = −0.10 μV, *SE* = 0.68), but the difference was not significant, *F*(1, 33) = 0.66, *p* = 0.42, *η*^2^ = 0.02 (see Figure 4). All other main effects and interactions were non-significant, all *F*s < 0.59, all *p*s > 0.56.

## 4. Discussion

The purpose of the study was to investigate whether short-term video game exposure influences the neural correlates of conflict detection and resolution as measured by the N2 and SP in a CStroop task. Based on the previous research with the genres of video games utilized in this study, it was hypothesized that exposure to UT would result in lower accuracy for incongruent trials, increased N2 amplitude, and decreased SP amplitude; in contrast, exposure to SC was expected to improve accuracy, decrease the amplitude of the N2, and increase the amplitude of the SP. The data did not reveal statistically significant group differences or effect sizes suggestive of a relationship between the video games and the measured outcomes. Post hoc power analyses indicated that the current sample was underpowered to detect group differences for at least three of the four dependent variables (accuracy: 39%; RT: 19.5%; N2: 23%; SP: 72%), suggesting caution when drawing conclusions from these data. The results are discussed in terms of interesting patterns worth further exploration and important limitations that should be addressed in future studies.

### 4.1. Behavioral Data

The behavioral data are consistent with previous work using a proportion congruent manipulation of the CStroop [18]. Overall, accuracy was lower and reaction times were slower for I compared to C trials, with accuracy for I trials being higher in the Mi block (i.e., expected conflict) compared to the Mc block. The Block by Congruency interaction demonstrates that I trials were responded to significantly faster in the Mi block than in the Mc block, and C trials were responded to significantly slower in the Mi block than in the Mc block. This indicates that the context of the block affects the two trial types differently, with participants responding faster on the trials that matched their expectation within each block. For accuracy, the Block by Congruency interaction demonstrates that participants responded with higher accuracy to I trials in the Mi block, suggesting that they handled the conflict trials more effectively when there were more of them. While participants do experience more conflict for I trials compared to C trials, the conflict is attenuated when they are expecting it to occur because most of the trials are high conflict. These results indicate that participants were sensitive to the probability of conflict.

Contrary to the hypotheses, accuracy did not differ significantly by video game group. A potential explanation for this is a ceiling effect; it is possible that the CStroop task was too easy, which led to all participants having relatively little trouble with the task, regardless of the game they were exposed to or the conflict they were experiencing. Evidence of a potential ceiling effect is seen in the relatively high accuracy scores for both groups. There is no condition that has an accuracy score of below 90%, which indicates that all participants completed the task with ease, possibly obscuring any effects of the games. A more challenging task may be able to better determine the effects of video game exposure on behavioral measures of cognitive control.

### 4.2. ERP Data

#### 4.2.1. N2

The N2 does not appear to be capturing the proportion congruent manipulation of the CStroop, contrary to past research demonstrating a difference between C and I trials in ERP components measuring conflict response for participants performing the CStroop [18]. Contrary to the hypothesis, there were no significant main effects or interactions of Game in the data. As the N2 reflects the detection of conflict, the results provide inconclusive information on the participants’ ability to detect conflict while completing the CStroop task after playing either a strategy game or an FPS game. One potential explanation for the N2 not matching the behavioral data or previous results is the nature of ERP components: an ERP component is only one process, but a single behavior or action is made up of multiple, potentially millions, of different processes happening simultaneously. It is possible that different ERP components are needed to observe the desired cognitive effects.

Further investigation of the data demonstrated an interesting pattern for the UT group that does support the hypothesis. Visually, the UT group appears to demonstrate the expected differences elicited by the proportion congruent manipulation in the N2, with the peak amplitudes slightly larger in response to I trials for all conditions, although these differences are not significant. The SC group, on the other hand, appears to have nearly identical N2 responses to all conditions. The effect size for the group difference was very small (*η*^2^ = 0.002), but given the post hoc power analysis, it may be worth examining this further with a larger sample or in a more challenging version of the task.

#### 4.2.2. SP

The results of the SP are consistent with the behavioral data and with the proportion congruent CStroop manipulation [18]. The significant Congruency effect demonstrates that participants had more difficulty with the I trials than the C trials, indicating that they experienced conflict in the I trials. The significant Block by Congruency interaction effect demonstrates that the I trials provoked a greater response than the C trials in the Mc/I condition, more so than in the Mi/I condition, indicating a significant difference between C and I trials. This is consistent with what would be expected in the different types of conflict, as the Mi block represents an expected conflict environment and should have a lesser difference in I and C trials because the participant should be more attenuated to the conflict. These results align with the significant Congruency effect and significant Block by Congruency interaction effect observed in accuracy and reaction time. As the SP reflects the resolution of conflict, the results for the SP in combination with the behavioral results suggest that participants had more difficulty resolving conflict when it was unexpected and more ease in resolving conflict when it was expected.

There are no significant differences between video gaming groups for the SP, which is inconsistent with the hypothesis. The non-significance of the SP data provides inconclusive results on the differences of the two video gaming groups in resolving conflict. A potential explanation for this, similar to the N2, could be the insensitivity of the ERP component in detecting the desired neural output. As mentioned previously, an ERP component is not sensitive to every interaction occurring in the brain at a single moment, and as each action is made up of millions of processes, this could limit the efficacy of using the SP in measuring the neural correlates associated with the effects of the video games.

Interestingly, the data indicate that the UT group has a slightly larger overall peak than the SC group. This pattern, while non-significant, suggests that the UT group had slightly more difficulty in resolving the conflict of the task than the SC group. Again, the small effect size (*η*^2^ = 0.02) warrants caution in interpreting the result, but the presence of this pattern, along with the significant Block by Congruency effect demonstrating an overall greater difficulty in resolving unexpected conflict, indicates that further research needs to be performed on the possibility of exposure to an FPS game affecting conflict resolution.

### 4.3. Limitations

It is worth discussing a few limitations of the current study that may have impacted the results. There was a limited participant pool, with participants drawn from a private, liberal arts college. The sample is not reflective of the entire population, so these results are not completely applicable to all populations. The results are skewed more towards women between the ages of 17 and 24, with middle to upper socioeconomic status (SES), who are attending college. In contrast, many of the previous studies on video gaming used as background for this study were performed with males as the majority of the participant pool [8,10,12,14,15,16]. It is possible that there are sex differences in the effects of video game exposure, so this difference in demographics could partially explain the inconsistency between the results of this study and previous studies. Furthermore, the participants were not asked to report race/ethnicity, so diversity in the sample is unknown. A more generalizable sample would have more men, more age ranges, multiple races/ethnicities, more diversity in SES, and a more equal distribution of genders.

In addition to the issues with sampling, there were also problems with the setup of the study that could have led to the lack of significant results. It is possible that the ERPs examined for this study were not sensitive to the experimental manipulation of this study. As discussed previously, an individual’s actions and cognition at any one moment are made up of an immense number of neural processes, and any single ERP only captures one or a small number of these processes. Furthermore, cognitive control is multi-faceted and video games may not impact all aspects of it [4]; the current study only examined conflict detection and resolution. The N2 and SP may not have been detecting the facets of cognitive control that were affected by the video game exposure. It is possible that the effects of the short-term video game exposure could have been detected by a different set of ERPs representing a different aspect of cognitive control such as inhibition or attention. Another issue with the setup of the study, also discussed previously, could be the level of difficulty of the CStroop task. Since no condition had an accuracy score below 90%, it is likely that there was a ceiling effect, suggesting that the participants of both groups were not challenged by the task, regardless of the presence of high conflict trials. The ease with which the task was completed could potentially diminish the effects of the video game exposure.

Finally, the marginal differences in ADHD and impulsivity scores between groups suggest that random assignment to a game condition did not completely control for preexisting differences. The differences between groups indicate that the SC group may have had higher levels of ADHD symptoms and impulsive tendencies in comparison to the UT group. This is relevant for the N2, as the SC group did not show any effect of the task. In order to test if this was relevant in the current study, ANCOVAs were performed on accuracy, RT, N2 and SP amplitude with ADHD and BIS-11 scores as covariates. The covariates were not significant (all *p’s* > 0.19) in any analysis, with the exception of ADHD in the N2 analysis (*p* = 0.03). However, there was still no significant difference between the groups (*p* = 0.47), indicating that the covariate did not adjust the association between game and N2 amplitude. Still, future studies may explore the potential relationship between N2 amplitude, ADHD, impulsivity, and gaming. It is possible that high levels of ADHD and impulsivity could cancel out the effects of playing a strategy game, as strategy games usually require careful planning, consideration of different possibilities, and attention to detail, while ADHD and impulsivity are characterized by difficulty concentrating, a lack of self-control, hyperactivity, and challenges in sustaining attention [26,27]. This idea is supported by a study that found that attention impulsiveness, measured by the BIS-11, was associated with an ineffectual and inefficient conflict detection process, measured by the N2 [26], as well as by another study that found that adolescents and teens with ADHD demonstrated decreased N2 responses in a cognitive control task, although the N2 response increased with age [27]. If an individual has personality traits that are opposite to the traits utilized in the game, it is possible that the game would produce no effect.

### 4.4. Future Research

As mentioned previously, there are a number of issues with the current study, such as a non-representative sample, ceiling effects, and the improper component for the experimental manipulation. Future research could address these issues by making a number of changes to the current study. First, to reconcile the non-representative sample, future studies should sample from a wider population, such as community groups outside of a college campus to include different ages and SES. Additionally, there should be a more even balance of males and females, and participants should be asked to record their racial identity. Second, to address the ceiling effect, the CStroop task could be made more challenging to elicit stronger responses to conflict. Third, the issue with the sensitivity of the ERPs could be addressed by analyzing ERPs that represent a different aspect of cognitive control such as error detection, task-switching, or attention and motivation. For example, measuring and analyzing the P3, which indexes attending to unfamiliar and rare stimuli [28], and the P1, which represents the direction of attention to stimuli [29], could demonstrate effects not significantly observed in the N2 and SP. It is possible that the video game manipulation of this study led to differences in cognitive processes better represented by different ERPs, which could be tested with this change in the study. Finally, an interesting future study could include a control group that would not be exposed to any video game. It is possible that there is a difference between short-term video game exposure and no video game exposure, which this study did not examine. The inclusion of a control group would allow for this possibility to be tested. Overall, future studies such as the ones described here could better examine the potential effects of short-term video game exposure on cognitive control.

## 5. Conclusions

The small effect sizes for between group differences in the three main outcomes (accuracy, N2, and SP) appear to indicate that 20 min of exposure to either of the video games used in this study has little impact on conflict detection or resolution in the CStroop task. However, given the post hoc power analyses and other limitations of the current study, the short-term effects of video games are worth further exploration. If the true effect size is very small, it may still be meaningful for individuals that play video games, so future studies may want to replicate this with a larger sample size. Additionally, stronger study designs employing pre–post testing, within subject manipulations, or control groups might be better suited to reveal the impact of short-term exposure to video games on cognitive control. The current study is a start to answering this question, and the authors hope that it will inspire others to explore the short-term effects of video games on cognition further.

## Data Availability

The data presented in this study are available on request from the corresponding author. The data are not publicly available due to university policy.
